# A study on the application of range shifter and bolus in spot-scanning proton arc (SPArc) therapy after modified radical mastectomy for left-sided breast cancer

**DOI:** 10.3389/fpubh.2026.1773124

**Published:** 2026-05-22

**Authors:** Linsen Zhou, Yiwen He, Shiyan Shen, Xiaobin Wang, Shuokai Jia, Hexiang Feng, Xinjian Yang, Xianhu Zeng

**Affiliations:** 1 Sichuan University, Chengdu, China; 2 Department of Radiotherapy Physics & Technology, West China Hospital, Sichuan University, Chengdu, Sichuan, China

**Keywords:** bolus, EDIC, left-sided breast cancer, NTCP, postoperative radiotherapy, range shifter, spot-scanning proton arc (SPArc) therapy

## Abstract

**Background:**

This study aimed to compare the dosimetric differences between the use of range shifter and bolus in spot-scanning proton arc (SPArc) therapy for postoperative radiotherapy after left-sided breast cancer modified radical mastectomy, with a primary focus on target volume dose coverage and organs-at-risk sparing. By comparison with VMAT, this study further explored the potential of SPArc to reduce the normal tissue complication probability (NTCP) and the effective dose to immune cells (EDIC).

**Methods:**

This retrospective study utilized the imaging datasets of 18 patients who treated with VMAT. Three SPArc plans were designed for each patient using the RayStation v2025: 1. SPArc with bolus (SPArcB); 2. SPArc with range shifter (SPArcR); 3. SPArc with both bolus and range shifter (SPArcRB). The target volumes included the supraclavicular target volume and the chest wall target volume. The Lyman–Kutcher–Birman NTCP model was adopted to evaluate pulmonary and cardiac toxicities and the EDIC model was applied to assess the immune cell dose across different plans. Robustness optimization accounting for a ± 3.5% range uncertainty and a 5 mm setup error was incorporated into all SPArc treatment plans.

**Results:**

All SPArc plans met robustness criteria and significantly reduced doses to the heart and left lung, as well as EDIC, compared to VMAT. The SPArcR plan demonstrated superior cardiac protection, achieving the lowest mean heart dose and the most significant reduction in NTCP for both the heart and lungs. While SPArcB showed a slight advantage in reducing the mean left lung dose, SPArcR yielded the highest conformity index for target coverage.

**Conclusion:**

This study confirms that, in postoperative radiotherapy for left-sided breast cancer after modified radical mastectomy, the standalone application of a range shifter can optimally reduce cardiac dose and the associated risk of complications while ensuring adequate target volume coverage, and it circumvents the issues of setup variability and hygiene concerns associated with the clinical use of bolus.

## 1 Introduction

According to the latest data from the World Health Organization (WHO), over 2.3 million women worldwide were diagnosed with breast cancer in 2022, with 670,000 dying from the disease. In 157 out of 185 countries, breast cancer is the most commonly diagnosed cancer among women. It is projected that by 2040, the annual number of new breast cancer cases globally will exceed 3 million (1). In the comprehensive management of breast cancer, radiotherapy is an indispensable component of post-operative treatment. It aims to maximise local disease control, minimise the risk of locoregional recurrence, and improve patients’ overall survival rates (2). However, for left-sided breast cancer, due to its anatomical proximity to the heart, there is an elevated risk of radio-induced cardiac injury and fatal cardiovascular events. Studies indicate that for every 1 Gy increase in the mean heart dose, the risk of major coronary events rises by 7.4% (3). Furthermore, radiotherapy may also induce complications such as radiation pneumonitis and radiation myelopathy, which can severely compromise the patient’s quality of life (4, 5). How to ensure adequate dose coverage to the target volume while minimising radiation dose to adjacent organs at risk, such as the heart and lungs, remains the central challenge in clinical practice.

Compared to photon radiotherapy, proton therapy offers a potential solution to achieve the aforementioned goal, leveraging its unique physical property known as the Bragg peak. As the proton beam deposits its maximum energy at a specific depth, its dose undergoes a rapid fall-off thereafter, thereby allowing for a significant reduction in the radiation dose to organs at risk such as the heart and lungs. The study by Tommasino et al. (6) demonstrated that, while achieving equivalent target coverage, intensity-modulated proton therapy (IMPT) significantly reduced the radiation dose to the heart and lungs compared to intensity-modulated radiotherapy (IMRT). Moreover, the predicted risks of skin toxicity and cardiopulmonary diseases were also lower. The spot-scanning proton arc (SPArc) therapy, first proposed by Ding et al. (7), is a novel, robust, and efficient optimization algorithm for proton therapy. In recent years, multiple studies have demonstrated that in the treatment of tumors such as oropharyngeal cancer (8), liver cancer (9), and esophageal cancer (10), SPArc exhibits significant dosimetric advantages compared to techniques like IMPT and volumetric modulated arc therapy (VMAT), and shows potential for further reducing normal tissue toxicity. The study by Chang et al. on left-sided breast cancer whole breast radiotherapy demonstrated that, compared to IMPT, SPArc can further reduce the radiation dose to healthy tissues and lower the normal tissue complication probability (NTCP) (11). These findings confirm the feasibility and potential clinical value of SPArc in breast cancer radiotherapy.

In proton therapy, to generate a proton beam suitable for treating superficial targets, a range shifter (RS) is typically employed to degrade the beam energy. An RS is a homogeneous plate, fixed to the snout at a certain distance from the patient’s body, commonly made of materials such as acrylonitrile butadiene styrene or polyethylene (12). For VMAT, the application of a bolus over the chest wall target is commonly employed to improve superficial dose uniformity. This ensures adequate dose delivery to the shallow layer of the chest wall target following modified radical mastectomy for breast cancer, thereby aiding in the prevention of postoperative recurrence (13). However, as a novel proton therapy optimization algorithm, whether SPArc requires the use of a bolus to modify the superficial dose distribution for the chest wall target following left-sided modified radical mastectomy, and whether employing a bolus or an RS can achieve a superior balance between target dose coverage and optimized distal organ sparing, remains a subject that has not been thoroughly investigated.

This study aims to systematically evaluate the dosimetric differences between using a bolus or an RS in SPArc for post-operative radiotherapy following left-sided modified radical mastectomy for breast cancer. These SPArc plans will be compared with clinically implemented VMAT plans, with a focus on target dose coverage, organs at risk (OARs) sparing, and specific dosimetric parameters. Furthermore, the study will investigate the potential of different planning strategies to reduce the NTCP and the effective dose to immune cells (EDIC). By elucidating the impact of these technical parameters on dose distribution, this research will provide a theoretical basis for the individualized clinical selection and protocol optimization of SPArc technology.

## 2 Materials and methods

### 2.1 Selection of retrospective patient data

This study protocol was approved by the Institutional Review Board of West China Hospital, Sichuan University (Approval No.: 2025227). All procedures were performed in accordance with local laws and institutional requirements. Written informed consent was obtained from all participants. The datasets generated and analyzed during this study are not publicly available due to privacy restrictions, but are available from the corresponding author upon reasonable request. This retrospective study consecutively included imaging datasets of patients who underwent radiotherapy following modified radical mastectomy for left-sided breast cancer at our institution between 2024 and 2025. Eighteen patients were ultimately enrolled, with no eligible patients excluded, thereby minimizing selection bias. The inclusion criteria were as follows: 1. Pathologically confirmed left-sided breast cancer and receipt of VMAT; 2. High risk of skin recurrence assessed by physicians, requiring the use of a bolus with consistent coverage of the chest wall target volume as prescribed during treatment; 3. Target volume encompassing both the chest wall and supraclavicular fossa; 4. Patient immobilization using a breast vacuum cushion, with treatment delivered under free breathing at a prescribed dose of 5,000 cGy in 25 fractions. Detailed characteristics of the patients and tumours are presented in Table 1.

**Table 1:** Patient and tumor characteristics.

Characteristic	Value
Patients	18
Age at RT (years)	55 (42–70)
Target volume (cc)	
CTVsc	120.98 (66.26–426.84)
CTVcw	405.85 (123.87–719.55)
Location	
Central breast	8 (44.44%)
Upper inner quadrant	2 (11.11%)
Upper outer quadrant	4 (22.22%)
Lower outer quadrant	4 (22.22%)
T stage	
T1	5 (27.78%)
T2	8 (44.44%)
T3	3 (16.67%)
T4	2 (11.11%)
N stage	
N0	4 (22.22%)
N1	12 (66.67%)
N2	1 (5.56%)
N3	1 (5.56%)
M stage	
M0	17 (94.44%)
M1	1 (5.56%)
Laterality	
Left	18

The datas of age at RT and target volume are presented as the median and range of all patients.

### 2.2 Patient setup, description of target volumes and OARs

All patients underwent patient immobilization using a vacuum cushion equipped with an oblique plate, with a bolus (Shenzhen Tongchuang Medical Technology Co., Ltd.) applied outside the chest wall target volume. The bolus coverage extended from the inferior border of the left clavicle to the inferior margin of the breast, medially to the anterior midline, and laterally to the midaxillary line. Target volumes and OARs were contoured slice by slice by physicians on simulation CT images. The clinical target volume (CTV) was defined as the supraclavicular lymph node CTV (CTVsc) and chest wall CTV (CTVcw), including residual glandular tissue, adjacent adipose tissue, surgical scars, skin, subcutaneous tissue, and intercostal muscles. The planning target volumes (PTVsc and PTVcw) for the VMAT plan were generated by expanding CTVsc and CTVcw by 0.5 cm, respectively. All PTVs and CTVs included the skin surface. The contoured OARs included the left lung, right lung, heart, left anterior descending (LAD) artery, spinal cord, contralateral breast, left humeral head, and thyroid gland. The OARs constraints for plan optimization are presented in Table 2.

**Table 2:** Dose constraints and planning priority for organs at risk.

Structure	Parameter	Optimal criteria	Priority
Heart	Mean dose (cGy)	<800 cGy	High
	V500 cGy (%)	<40%	High
LAD	Mean dose (cGy)	<2,500 cGy	High
	V4000 cGy (%)	<20%	High
Lung_L	Mean dose (cGy)	<1,400 cGy	High
	V500 cGy (%)	<50%	High
	V1000 cGy (%)	<35%	High
	V2000 cGy (%)	<25%	High
Spinal_Cord	Max dose (cGy)	<2,500 cGy	High
Thyroid	Mean dose (cGy)	<2,800 cGy	Middle
Lung_R	V500 cGy (%)	<20%	Middle
Breast_R	Mean dose (cGy)	<400 cGy	Low
Humerus_Head_L	Max dose (cGy)	<5,000 cGy	Low
	V3000 cGy (%)	<20%	Low

V500 cGy, V1000 cGy, V2000 cGy, V3000 cGy, and V4000 cGy refer to the percentage of the volume of the respective organs that receives at least the corresponding dose, respectively.

### 2.3 Dose prescription, treatment planning, and robust optimization

The prescribed dose to the target volume was 5,000 cGy in 25 fractions. The treatment plan was designed to ensure that at least 98% of the CTV receives 100% of the prescribed dose, while the maximum dose within the target volume does not exceed 107% of the prescribed dose. The VMAT treatment plan was optimised based on the PTV, whereas the SPArc treatment plan was optimised directly on the CTV. A constant relative biological effectiveness (RBE) value of 1.1 was adopted in the proton therapy plan (14). To minimise potential biases from different treatment planning systems (TPS), all treatment plans were calculated and generated using RayStation v2025 (RaySearch Laboratories AB, Stockholm, Sweden). For each patient, four radiotherapy plans were designed using SPArc and VMAT techniques, respectively (Table 3). The bolus was modeled as a water-equivalent material (density = 1.0 g/cm^3^) with a physical thickness of 0.5 cm, corresponding to a water-equivalent thickness of 0.5 cm. This thickness increases superficial dose to ensure adequate target coverage, consistent with routine clinical practice. In the no-bolus group, the corresponding region was assigned as air to simulate the clinical scenario without the use of a bolus. The RS was modeled as polymethyl methacrylate (PMMA) with a density of 1.19 g/cm^3^, a physical thickness of 3.65 cm, and a field size of 30 × 40 cm^2^. This thickness shifts the proton beam range forward to the required depth for adequate superficial target coverage. PMMA was selected as the RS material due to its near water-equivalent stopping power and well-characterized scattering properties, which help ensure dose calculation accuracy in the treatment planning system. Although PMMA introduces slightly greater scattering compared with materials such as paraffin or polyethylene, this effect is clinically acceptable (15). In addition, PMMA has stable density, high mechanical strength, and resistance to deformation, making it suitable for clinical use, and it is the default range shifter material for the IBA ProteusPlus proton therapy system.

**Table 3:** Radiation therapy plan setup.

Groups	Bolus materials	Range shifter	Abbreviation
VMAT with Bolus	Water	NO	VMAT
SPArc with Bolus without RS	Water	NO	SPArcB
SPArc without Bolus with RS	Air	YES	SPArcR
SPArc with Bolus with RS	Water	YES	SPArcBR

Bolus materials and RS are both set up in the RayStation v2025. VMAT with bolus is abbreviated as VMAT; SPArc with bolus without RS is abbreviated as SPArcB; SPArc without bolus with RS is abbreviated as SPArcR; SPArc with bolus with RS is abbreviated as SPArcBR.

For the SPArc plan, a single arc discretized into 20 directions was used. Each plan consisted of 360 energy layers, and the spot positions were automatically determined by the treatment planning system based on the patient’s individual anatomy. Delivery constraints were applied, with the minimum and maximum monitor units per spot set to 0.01 MU/fraction and 15 MU/fraction, respectively. The SPArc plans were optimized using the Monte Carlo algorithm, with the proton beam model based on the IBA ProteusPlus system. The proton beam energy ranged from 70 to 227 MeV, with spot sizes ranging from 2.73 mm (at 227 MeV) to 7.0 mm (at 70 MeV). For the VMAT plan, dual arcs were generated using 6 MV photon beams. The gantry angle spacing was set to 2°, and the maximum delivery time was limited to 120 s. The collimator angle was set to 0° for all plans. All plans utilized a single-isocenter tangential field arrangement.

All SPArc plans in this study underwent robustness optimization accounting for ± 3.5% range uncertainty and ± 5 mm setup uncertainty in six directions (anterior–posterior, posterior–anterior, left–right, right–left, superior–inferior, and inferior–superior). A total of 21 uncertainty scenarios, including nominal and perturbed conditions, were evaluated using a scenario-based worst-case analysis approach. For robustness evaluation, target coverage was quantified using *D*_95%_ for both CTVcw and CTVsc across all uncertainty scenarios. A plan was deemed robust if, in at least 80% of the scenarios, the *D*_95%_ for each target volume was no less than 95% of the prescribed dose. In addition, worst-case scenario analysis was performed to ensure that no clinically unacceptable target underdosage occurred under extreme perturbations (16).

### 2.4 Dose–Volume Histogram analysis

Dosimetric parameters of CTVsc and CTVcw were obtained via Dose–Volume Histogram (DVH) analysis to assess and compare various radiotherapy plans. In accordance with ICRU Report 83, dosimetric parameters including *D*_98%_, conformity index (CI), and homogeneity index (HI) were adopted in this study to evaluate target volume coverage. The calculation formulas for CI and HI are as follows (Equations 1,2):

$$\mathrm{CI}=\frac{V_{t,\mathrm{ref}}^{2}}{V_{t}\times V_{\mathrm{ref}}}$$ (1)

$$\mathrm{HI}=\frac{D_{2\%}-D_{98\%}}{D_{50\%}}$$ (2)

Where *V*_*t*,ref_ is the volume of the target volume covered by the prescribed dose, *V_t_* is the target volume, and *V*_ref_ is the volume of the reference isodose (5,000 cGy) within the target volume. *D*_2%_, *D*_50%,_ and *D*_98%_ are the minimum absorbed doses received by 2, 50, and 98% of the target volume, respectively. A higher CI indicates better dose conformity to the target volume, with an ideal value of 1; whereas a lower HI denotes a more uniform radiation dose distribution within the target, with an optimal value of 0 (17). To evaluate the protection of OARs, dosimetric parameters including mean dose, max dose, V500 cGy, and V2000 cGy were recorded. V500 cGy and V2000 cGy refer to the percentage of the volume of the respective organs that receives at least 500 cGy and 2000 cGy, respectively. Specifically: the heart was primarily evaluated by mean dose and V500 cGy; the left lung by mean dose, V500 cGy, and V2000 cGy; the right lung by mean dose and V500 cGy; the spinal cord by max dose as the core endpoint; the LAD by mean dose; the contralateral breast by mean dose; the left humeral head by max dose; and the thyroid gland by mean dose.

### 2.5 Evaluation of potential clinical benefit for OARs based on the NCTP model and hematological toxicity based on the EDIC model

The SPArc and VMAT plans developed for each patient will be evaluated for potential clinical benefits using the NTCP model. The Lyman–Kutcher–Birman NTCP model was adopted to calculate pulmonary and cardiac toxicity of different plans based on the mean dose. The definition of this model is as follows (Equations 3,4):

$$\text{NTCP}=\frac{1}{\sqrt{2\pi }}\int_{-\infty }^{t}e^{-\frac{x^{2}}{2}}dx$$ (3)

$$t=\frac{\mathrm{EUD}-{\mathrm{TD}}_{50}}{m\cdot {\mathrm{TD}}_{50}}$$ (4)

NTCP is determined by two parameters: TD₅₀ (tolerance dose for 50% complication), defined as the dose that results in a 50% complication rate when the entire organ is uniformly irradiated; and *m*, the slope parameter of the sigmoid dose–response curve, where a smaller value indicates a steeper curve. For assessing the risk of radiation pneumonitis, the equivalent uniform dose (EUD) was set as the mean lung dose, with TD₅₀ = 30.8 Gy and *m* = 0.37, based on the NTCP model proposed by Seppenwoolde et al. (18). For evaluating the risk of any grade of pericardial effusion, the EUD was adopted as the mean heart dose, with TD₅₀ = 34.3 Gy and *m* = 0.75, in accordance with the NTCP model developed by Fukada et al. (19). To compare the risk differences between each plan and the VMAT plan, the NTCP ratio was used, calculated as follows: NTCP ratio = NTCPSPArc/NTCPVMAT. This ratio was computed separately for SPArcB, SPArcR, and SPArcRB plans.

We adopted the EDIC model proposed by Jin et al. (20) to evaluate the dose to immune cells across different treatment plans. This model quantifies the cumulative radiation dose received by systemically circulating lymphocytes during radiotherapy. A study by Chen et al. has confirmed that the EDIC is an important factor influencing radiation-induced lymphopenia (RIL) in breast cancer radiotherapy, and can be used for individualised risk prediction via NTCP modellin (21). The definition of this model is as follows (Equation 5):

$$\begin{array}{l} \text{EDIC}=0.12\times \mathrm{MLD}+0.08\times \mathrm{MHD}+\\ \left[0.45+0.35\times 0.85\times \sqrt{\frac{n}{45}}]\times (\frac{\text{ITDV}}{61.8\times 10^{3}}\right) \end{array}$$ (5)

Where MLD refers to the mean lung dose; MHD denotes the mean heart dose; *n* represents the number of radiotherapy fractions; and ITDV stands for the integral total dose volume, defined as the total integral dose within the planning CT images divided by the average volume to obtain the mean dose.

### 2.6 Statistics

Statistical analyses were performed using IBM SPSS 27.0 (IBM Corp., Armonk, NY, USA). First, the Friedman test was employed to compare the overall differences among the four radiotherapy plans, with a *p*-value <0.05 indicating statistically significant differences. Where a significant overall difference was identified, pairwise comparisons were conducted using the Wilcoxon signed-rank test, supplemented by Bonferroni multiple testing correction. The corrected significance level was set at *α*’ = 0.00833.

## 3 Results

SPArc was used to design radiotherapy plans for 18 patients undergoing post-radical mastectomy radiotherapy for left-sided breast cancer, resulting in the generation and analysis of 72 treatment plans. The SPArcB plan (with the minimum air gap) had a mean air gap of 31.18 cm, whereas the SPArcR plan had a mean air gap of 27.74 cm, representing an average reduction in air gap of 11.03% (95% confidence interval: 10.03–12.03%). The SPArcRB plan had a mean air gap of 27.68 cm, corresponding to an average reduction in air gap of 11.01% (95% confidence interval: 10.07–11.96%).

### 3.1 Robustness analysis of different SPArc plans

Table 4 summarizes the robustness evaluation results of target coverage for CTVcw and CTVsc under all uncertainty scenarios. All three SPArc plans (SPArcB, SPArcR, and SPArcRB) satisfied the robustness evaluation criteria under 3.5% range uncertainty and 5 mm setup uncertainty, defined such that in at least 80% of the scenarios, the *D*_95%_ for each target volume was no less than 95% of the prescribed dose. For CTVcw, the mean *D*_95%_ values were comparable across plans, ranging from 5022.1 ± 47.6 cGy (SPArcB) to 5052.2 ± 31.7 cGy (SPArcRB), with SPArcR showing an intermediate value of 5038.8 ± 24.5 cGy. The worst-scenario *D*_95%_ values followed a similar pattern: SPArcB had the lowest mean worst-scenario value (4954.9 ± 57.7 cGy), while SPArcR and SPArcRB achieved slightly higher values (4988.6 ± 32.0 cGy and 4982.5 ± 32.3 cGy, respectively). For CTVsc, SPArcR demonstrated the most favorable robustness, with the highest mean *D*_95%_ (5039.1 ± 15.4 cGy) and the highest worst-scenario *D*_95%_ (4994.8 ± 22.5 cGy). In contrast, SPArcB exhibited the lowest mean *D*_95%_ (4989.9 ± 39.4 cGy) and notably the lowest worst-scenario *D*_95%_ (4867.6 ± 93.1 cGy), accompanied by the largest standard deviation, indicating greater sensitivity to uncertainties. SPArcRB showed intermediate robustness for CTVcw. Overall, while all plans remained within clinically acceptable limits, SPArcR provided the most robust target coverage, particularly for the CTVcw structure.

**Table 4:** Robustness analysis of target coverage (*D*_95%_) for CTVcw and CTVsc across all uncertainty scenarios.

Plan	Structure	Mean *D*_95%_ (cGy)	Worst scenario *D*_95%_ (cGy)
SPArcB	CTVcw	5022.1 ± 47.6	4954.9 ± 57.7
	CTVsc	4989.9 ± 39.4	4867.6 ± 93.1
SPArcR	CTVcw	5038.8 ± 24.5	4988.6 ± 32.0
	CTVsc	5039.1 ± 15.4	4994.8 ± 22.5
SPArcRB	CTVcw	5052.2 ± 31.7	4982.5 ± 32.3
	CTVsc	5026.4 ± 16.3	4965.6 ± 24.1

Values are presented as mean ± standard deviation (SD). “Mean *D*_95%_” is the overall mean of each patient’s average *D*_95%_ across scenarios; “Worst scenario *D*_95%_” is the overall mean of each patient’s minimum *D*_95%_ across scenarios. CTVsc: supraclavicular lymph node CTV; CTVcw: chest wall CTV.

### 3.2 Comparison of target volume dosimetry

**Figure 1:**
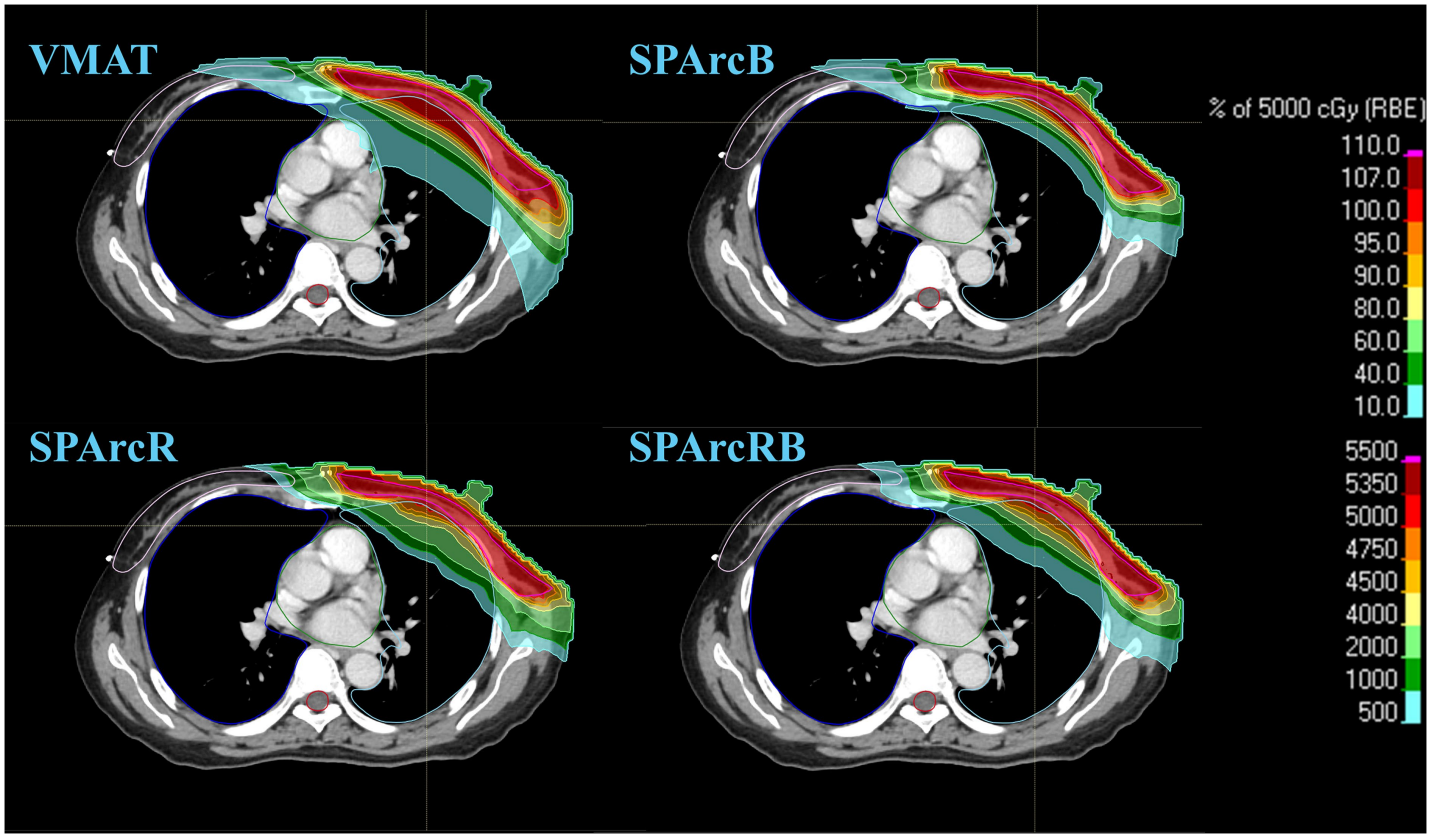
A representative of the radiation treatment plan. The comparison of patient dose distribution of CTVcw among four treatment plans (VMAT, SPArcB, SPArcR, SPArcRB).

The study demonstrated that different radiotherapy techniques (SPArc vs. VMAT) and parameter configurations (with or without bolus or RS) exerted a significant impact on the dose distribution of target volumes (*p* < 0.05). Figure 1 displays representative CT slices of one patient, presenting a comparison of the dose distributions of the CTVcw across the four radiotherapy plans. Among these plans, the SPArc plans exhibited markedly smaller low-dose regions (500 cGy, 1,000 cGy) than the VMAT plan, and the prescribed dose (5,000 cGy) was more conformal compared with that of the VMAT plan. According to Table 5, pairwise comparisons of the CI values of the four radiotherapy plans were performed using the Wilcoxon signed-rank test for the CTV as a whole. The results indicated that statistically significant differences existed between all pairs of plans. The CI values were ranked in descending order as follows: SPArcR > SPArcBR > SPArcB > VMAT. Specifically, SPArcR yielded the highest CI value, with the most significant difference observed relative to VMAT (*p* < 0.001). For the CTVsc, the *D*_98%_ and *D*_95%_ values of the VMAT plan were significantly higher than those of the SPArc plans, whereas no significant differences were observed among the SPArcB, SPArcR, and SPArcRB groups. The median *D*_98%_ values of all treatment plans reached the prescribed dose. In terms of the HI, the values of the SPArcB, SPArcR, and SPArcRB plans were significantly higher than that of the VMAT plan, with the most significant difference detected between the SPArcRB and VMAT plans. The target volume homogeneity of the VMAT plan was slightly superior to that of the SPArc plans; however, the HI values of all groups remained at a low level overall. For the CTVcw, the *D*_98%_ and *D*_95%_ values of the VMAT plan were lower than those of the SPArc plans, with a statistically significant difference observed between the VMAT plan and the SPArcRB plan. Specifically, the SPArcRB plan delivered the highest *D*_98%_ and *D*_95%_ values for the chest wall target volume, whereas no significant differences were detected among the SPArcB, SPArcR, and SPArcRB groups. In terms of the HI, no significant differences were found in pairwise comparisons of all the plans. The SPArcB group exhibited relatively high HI values, while the HI values of the SPArcR and SPArcRB groups remained at a low level.

**Table 5:** The dosimetric parameters and statistical results of target volumes and OARs.

Structure	Value	VMAT	SPArcB	SPArcR	SPArcRB	*p*	Pairwise comparisons
		Median (IQR)	Median (IQR)	Median (IQR)	Median (IQR)		
CTV	CI	0.574 (0.531–0.602)	0.608 (0.589–0.651)	0.718 (0.675–0.771)	0.676 (0.648–0.729)	<0.05	a,b,c,d,e,f
CTVsc	*D*_98%_ (cGy)	5065.72 (5053.52–5075.85)	5028.31 (5014.05–5043.28)	5023.45 (5007.67–5033.43)	5021.58 (5018.12–5039.62)	<0.05	a,b,c
	*D*_95%_ (cGy)	5092.62 (5072.90–5101.43)	5059.51 (5045.83–5079.71)	5062.32 (5041.76–5076.53)	5064.82 (5048.37–5074.98)	<0.05	a,b,c
	HI	0.052 (0.047–0.055)	0.059 (0.055–0.063)	0.062 (0.057–0.065)	0.063 (0.060–0.066)	<0.05	a,b,c
CTVcw	*D*_98%_ (cGy)	5022.94 (4987.54–5040.15)	5041.83 (5027.43–5057.33)	5031.00 (5003.57–5042.03)	5060.08 (5035.27–5069.95)	<0.05	c
	*D*_95%_ (cGy)	5065.44 (5056.04–5074.30)	5081.80 (5073.05–5093.83)	5079.01 (5054.45–5085.12)	5101.78 (5085.29–5111.58)	<0.05	c
	HI	0.060 (0.054–0.066)	0.061 (0.058–0.067)	0.057 (0.054–0.062)	0.055 (0.050–0.060)	<0.05	N/A
Heart	Mean dose (cGy)	426.04 (362.84–443.96)	145.19 (76.55–171.29)	80.17 (61.56–87.77)	98.61 (59.68–122.11)	<0.05	a,b,c,d,e
	V500 cGy (%)	14.32 (11.31–16.05)	6.53 (3.44–8.67)	3.89 (2.79–5.01)	5.19 (2.19–7.14)	<0.05	a,b,c,d
Lung_L	Mean dose (cGy)	1298.84 (1277.94–1345.74)	874.28 (792.98–972.49)	877.92 (829.09–913.57)	910.41 (867.07–966.73)	<0.05	a,b,c
	V500 cGy (%)	48.03 (46.93–50.32)	31.82 (29.67–34.84)	33.89 (32.14–35.40)	35.15 (33.67–35.97)	<0.05	a,b,c,e
	V2000 cGy (%)	23.45 (23.00–24.47)	17.75 (15.73–20.22)	18.38 (17.16–19.16)	18.86 (17.98–20.42)	<0.05	a,b,c,e
Lung_R	Mean dose (cGy)	197.96 (182.49–228.90)	17.64 (10.27–47.64)	20.29 (19.43–21.16)	33.97 (18.37–45.81)	<0.05	a,b,c,f
	V500 cGy (%)	4.80 (2.66–6.52)	0.43 (0.11–1.05)	0.19 (0.07–0.45)	0.84 (0.14–1.40)	<0.05	a,b,c,f
Spinal_Cord	Max dose (cGy)	804.76 (748.82–970.68)	98.39 (63.81–291.28)	152.78 (90.70–206.27)	328.18 (119.37–386.22)	<0.05	a,b,c,e,f
LAD	Mean dose (cGy)	1701.11 (1547.73–1978.14)	801.66 (529.25–995.39)	618.76 (538.29–701.98)	742.87 (486.20–850.49)	<0.05	a,b,c,e
Breast_R	Mean dose (cGy)	458.17 (288.12–473.83)	131.83 (91.64–161.31)	129.70 (101.22–168.51)	156.77 (124.82–184.13)	<0.05	a,b,c,f
Humerus_Head_L	Max dose (cGy)	4125.32 (3656.58–4412.14)	1798.56 (1516.40–2679.15)	2452.30 (1983.75–2777.64)	2397.37 (2028.59–3033.52)	<0.05	a,b,c,d,e
Thyroid	Mean dose (cGy)	2820.58 (2638.48–2941.30)	2092.50 (1983.77–2148.13)	2053.27 (2026.26–2174.85)	2275.15 (2145.27–2400.19)	<0.05	a,b,c,e,f

Data were presented as median values with interquartile ranges (IQR, Q1–Q3). The *p*-value is the result of the Friedman test between the four radiotherapy plans. *p* < 0.05 means there is statistically significant difference among the groups. “Pairwise comparisons” in the table use the Wilcoxon signed-rank test with Bonferroni multiple testing correction, and the corrected significance level was set at *α*’ = 0.00833. Pairwise comparison groups represented by the different letters. a: VMAT vs SPArcB;b: VMAT vs SPArcR;c: VMAT vs SPArcRB;d: SPArcB vs SPArcR;e: SPArcB vs SPArcRB; f: SPArcR vs SPArcRB.

### 3.3 Dosimetric analysis of organs at risk

**Figure 2:**
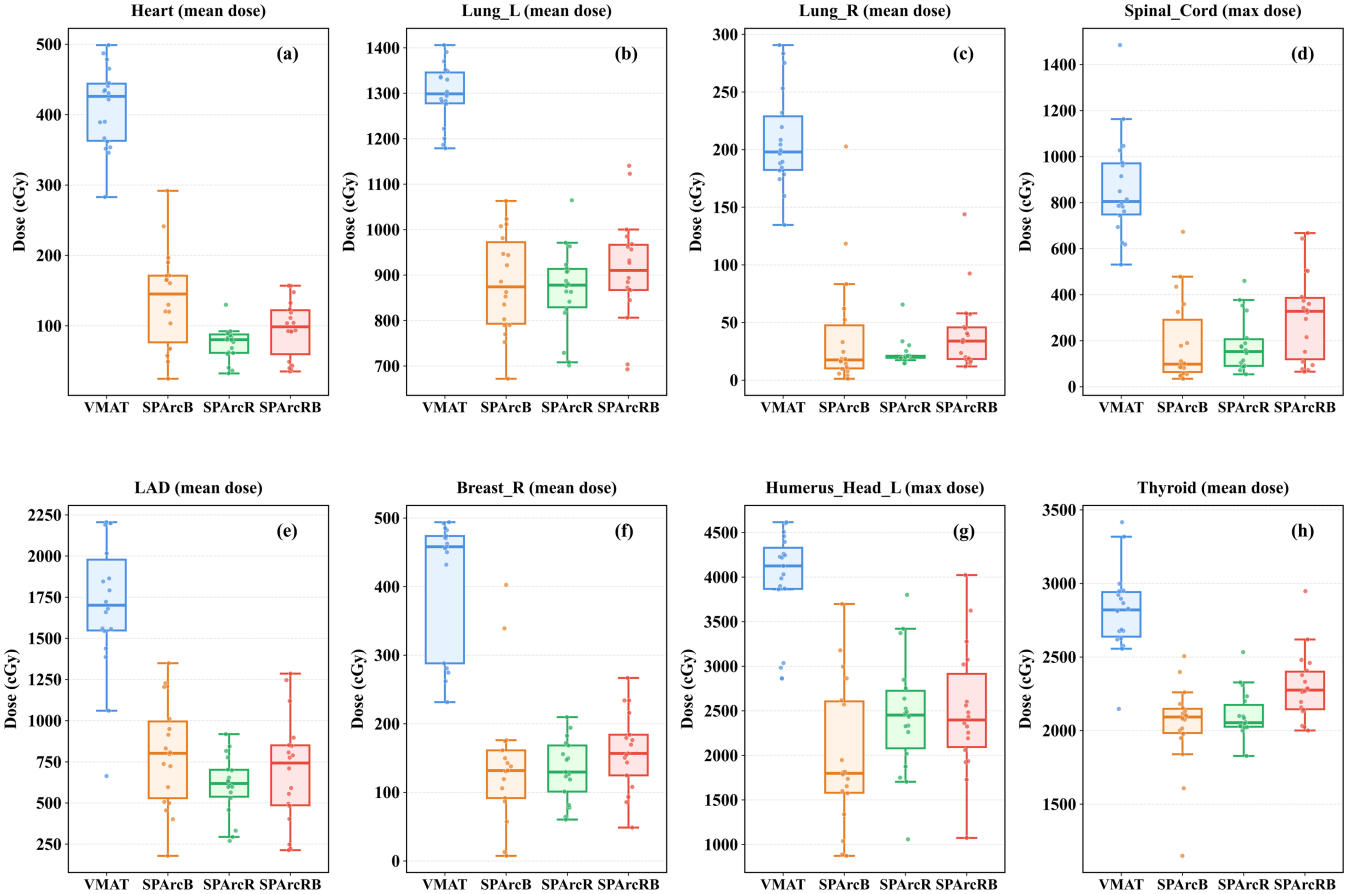
Box-and-whisker plots and scatter distribution plots of OARs among the four treatment plans. **(a)** mean dose to the heart; **(b)** mean dose to the left lung; **(c)** mean dose to the right lung; **(d)** max dose to the spinal cord; **(e)** mean dose to the LAD; **(f)** mean dose to the right breast; **(g)** max dose to the left humerus head; **(h)** mean dose to the thyroid.

As shown in Figure 2, SPArc significantly reduced the mean heart dose, with a further reduction observed following the addition of an RS. The median mean heart dose of SPArcR was the lowest (80.17 cGy), representing an 81.18% reduction compared with the highest value recorded for VMAT (426.04 cGy) (*Δ* = 345.87 cGy). A statistically significant difference was noted between SPArcR and both SPArcB and SPArcRB; the median mean heart dose of SPArcR (80.17 cGy) was 44.78% lower than that of SPArcB (145.19 cGy) (*Δ* = 65.02 cGy). The cardiac V500 cGy exhibited an identical trend: the V500 cGy of SPArcR was the lowest (3.89%), showing a 72.84% reduction relative to the highest value of VMAT (14.32%) and a 40.43% reduction compared with SPArcB (6.53%). RS demonstrated a significantly superior protective effect on the heart compared with bolus. For the left lung, the mean dose to the left lung delivered by SPArc plans was significantly lower than that delivered by the VMAT plan. Among these, the median mean dose of SPArcB was the lowest (874.28 cGy), representing a 32.69% reduction compared with the highest value of the VMAT plan (1298.84 cGy) (*Δ* = 424.56 cGy). No significant differences were observed among the SPArcB, SPArcR, and SPArcRB groups. The V500 cGy and V2000 cGy values of the left lung exhibited an identical trend: the median values of SPArcB were the lowest, and a statistically significant difference was detected between SPArcB and SPArcRB (*p* = 0.007). Analysis of the mean dose to the right lung showed that the median mean dose of SPArcB was the lowest (17.64 cGy), representing a 91.10% reduction compared with that of VMAT (197.96 cGy) (*Δ* = 180.32 cGy). No significant difference was observed between SPArcR and SPArcB, and SPArcR yielded the lowest median V500 cGy for the right lung. For the spinal cord, the median maximum dose of SPArcB was the lowest (98.39 cGy), which was 87.77% lower than that of VMAT (804.79 cGy) (*Δ* = 706.40 cGy). Both SPArcR and SPArcB had relatively low median maximum doses with no statistically significant difference between them, and both were significantly superior to SPArcRB. Analysis of the mean dose to the LAD revealed that SPArcR had the lowest median value (618.76 cGy), a reduction of 63.63% compared with VMAT (1701.11 cGy) (*Δ* = 1082.35 cGy). Moreover, a significant difference was found between SPArcR and SPArcB, with the dose of SPArcR being 22.82% lower than that of SPArcB (801.66 cGy) (*Δ* = 182.90 cGy). Compared with VMAT, all SPArc plans effectively reduced the mean doses to the thyroid gland and the right breast, with SPArcR achieving the greatest reduction. Regarding the median maximum dose to the left humeral head, the SPArcB group had the lowest value (1798.56 cGy), a decrease of 56.40% compared with VMAT (4125.32 cGy) (*Δ* = 2326.76 cGy).

### 3.4 Comparison of NTCP and EDIC

We adopted the Lyman-Kutcher-Birman NTCP model to calculate the pulmonary and cardiac toxicities of different treatment plans, so as to evaluate the risk of radiation pneumonitis and the risk of pericardial effusion of any grade associated with each plan. In addition, the EDIC model was used to compute the immune cell dose for the four radiotherapy plans. The results are presented in Table 6.

**Table 6:** Comparison of NTCP ratio for heart and lung as well as EDIC among four treatment regimens.

NTCP				
OARs	Clinical endpoint	SPArcB (NTCP_SPArcB_/NTCP_VMAT_)	SPArcR (NTCP_SPArcR_/NTCP_VMAT_)	SPArcRB (NTCP_SPArcRB_/NTCP_VMAT_)
Heart	pericardial effusion	0.835 (0.747–0.930)	0.792 (0.757–0.844)	0.806 (0.744–0.857)
Lung	radiation pneumonitis	0.536 (0.384–0.671)	0.510 (0.426–0.593)	0.553 (0.420–0.655)

EDIC(Gy)			
VMAT	SPArcB	SPArcR	SPArcRB
2.698 (2.045–3.067)	1.569 (1.203–2.159)	1.574 (1.105–1.801)	1.699 (1.064–1.903)

Data were presented as the median and range.

**Figure 3:**
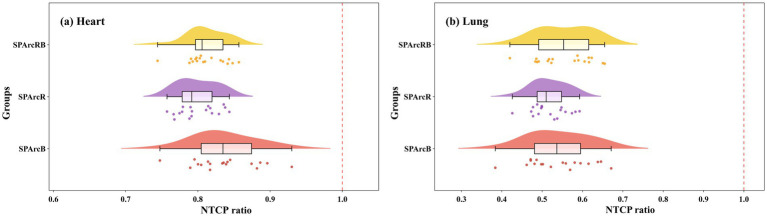
Comparison of NTCP ratios for heart and lung across different treatment plans (NTCP ratio = NTCP_SPArc_/NTCP_VMAT_). The vertical axis indicates the grouping, and the horizontal axis represents the NTCP ratio (NTCP_SPArc_/NTCP_VMAT_), where a value of 1 indicates equal NTCP between the two regimens. The width of the upper part of the violin plot represents the density of data at the corresponding values. A wider area indicates a higher density of data points within that range. The middle section displays a box plot. The lower section shows a scatter plot. **(a)** Presents the risk of pericardial effusion of any grade, while **(b)** shows the risk of radiation pneumonitis.

As shown in Figure 3, SPArc achieved an overall reduction in the predicted risks of pulmonary and cardiac toxicity compared with VMAT. According to the model calculations with pericardial effusion as the endpoint, the SPArcR yielded the most substantial reduction (median NTCPSPArcR/NTCPVMAT = 0.792), followed by the SPArcRB, while the SPArcB showed the smallest reduction. The model calculations with radiation pneumonitis as the endpoint also indicated that the SPArcR had the largest reduction (median NTCPSPArcR/NTCPVMAT = 0.510), followed by the SPArcB, whereas the SPArcRB exhibited the smallest reduction. Overall, the reduction in pulmonary NTCP achieved by SPArc was greater than that in cardiac NTCP, and the RS demonstrated superior efficacy in reducing both cardiac and pulmonary NTCP.

**Figure 4:**
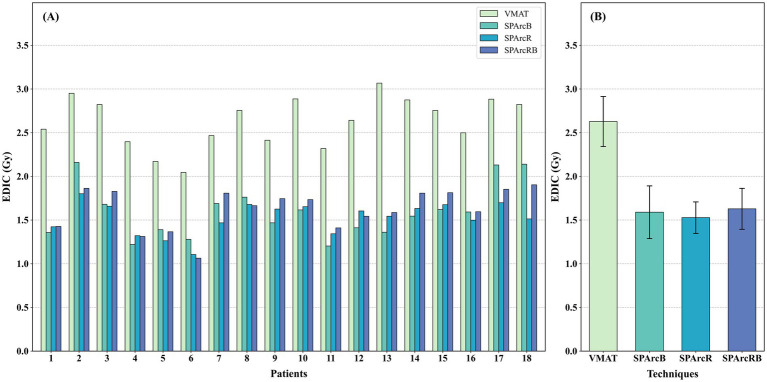
EDIC(Gy) for all patients across different treatment plans. **(A)** Bar chart showing individual EDIC values for each patient across the four treatment plans (VMAT, SPArcB, SPArcR, and SPArcRB). The height of each bar represents the absolute EDIC value. **(B)** Bar chart showing the mean EDIC values for the four treatment plans. The height of each bar represents the mean EDIC value of the respective group, and the error bars indicate the mean ± standard deviation.

The EDIC analysis results revealed that SPArc could substantially reduce the EDIC values (Figure 4). Among these plans, the SPArcB had the lowest median EDIC value (1.569 Gy), representing a 41.58% reduction compared with the VMAT plan (2.698 Gy) (*Δ* = 1.129 Gy). The median EDIC value of the SPArcR was slightly higher than that of the SPArcB (SPArcR = 1.574 Gy vs. SPArcB = 1.569 Gy, *Δ* = 0.005 Gy), with no statistically significant difference observed in pairwise comparisons. The SPArcRB had the highest median EDIC value among all SPArc plans; however, its value remained significantly lower than that of the VMAT plan, demonstrating the potential of SPArc technology to preserve lymphocytes. As shown in Figure 4B, SPArcR exhibited the lowest mean EDIC value and the smallest standard deviation, followed by SPArcB and SPArcRB. The mean EDIC values of all three SPArc plans were significantly lower than that of VMAT, indicating that SPArcR provides superior average protection with less inter-individual variability and more stable efficacy. Overall, all SPArc techniques exhibit significant lymphocyte-sparing potential compared with VMAT.

## 4 Discussion

To our knowledge, this is the first comprehensive dosimetric planning study designed to systematically evaluate and compare the impact of bolus versus range shifter settings on target and OARs dosimetric parameters, as well as NTCP and EDIC, for both SPArc and VMAT techniques in postoperative radiotherapy for left-sided breast cancer following modified radical mastectomy. Our results demonstrate that all SPArc plans can provide superior OARs sparing while maintaining target dose coverage compared to VMAT plans, showing potential protective advantages for the heart, lungs, and circulating immune cells.

Our analysis primarily focused on the effects of bolus and RS application on SPArc plans. We found that the use of RS yielded better target volume conformity for the CTV as a whole. This could be attributed to the fact that, for superficial target volumes such as the chest wall, the range of the minimum proton beam energy exceeds the target depth. RS can effectively reduce beam energy and better regulate the distribution of Bragg peaks, thereby enabling full-dose modulation at the patient’s surface. This also provides a potential avenue for subsequent linear energy transfer (LET) optimization based on variable RBE models (22). Notably, in this study, VMAT plans were optimized on the PTV, whereas SPArc plans were optimized directly on the CTV with robust optimization. For VMAT, the standard approach is to expand the CTV to a PTV and optimize on the PTV to account for setup and motion uncertainties. In proton therapy, however, because dose distributions are highly sensitive to range and setup uncertainties, robust optimization performed directly on the CTV is commonly adopted to explicitly address these uncertainties, rather than relying solely on geometric margin expansion. Therefore, although the two techniques differ in their optimization volumes, each conforms to its respective standard of clinical practice. We consider this comparison to be clinically relevant and do not anticipate that it introduces substantial bias into the main conclusions of this study.

The robustness analysis confirmed that all SPArc plans met the predefined criteria under ± 3.5% range and ± 5 mm setup uncertainties, indicating adequate target coverage across scenarios. However, differences in robustness were observed. For CTVcw, worst-case *D*_95%_ values suggested slightly improved stability in SPArcR and SPArcRB compared with SPArcB. For CTVsc, SPArcR demonstrated the most favorable robustness, with higher worst-case *D*_95%_ and lower variability, whereas SPArcB showed greater sensitivity to uncertainties. Overall, although all plans were clinically acceptable, SPArcR provided more consistent target coverage under uncertainty, particularly in worst-case conditions.

For dosimetric indices of the CTVsc and CTVcw, including *D*_98%_, *D*_95%_ and HI, no statistically significant differences were observed between the SPArcR and SPArcB plans, with all indices meeting the prescribed dose requirements. This indicated that both bolus and RS configurations could ensure adequate target volume coverage. In terms of OARs sparing, the use of RS conferred prominent protective effects on the heart: it delivered the lowest median mean cardiac dose and V500 cGy, thereby achieving the maximum reduction in the risk of pericardial effusion of any grade. Furthermore, RS also exhibited significantly superior protective efficacy for the left LAD compared with other plans. For all OARs except the left humeral head, no statistically significant differences were detected between the bolus and RS configurations, with both significantly outperforming the VMAT plan currently implemented in clinical practice. In the NTCP prediction for radiation pneumonitis, RS yielded the greatest reduction in the risk of this complication. In the EDIC analysis, no significant differences were found between the bolus and RS configurations, which could be attributed to the excellent protective effects of SPArc technology on both the heart and lungs. A study by Chen F et al. involving 735 breast cancer patients demonstrated a significant correlation between EDIC and RIL. The EDIC value corresponding to a 50% incidence of grade 1 RIL was 1.2 Gy, while the EDIC values associated with a 50% incidence of grade 2 and grade 3 RIL were 2.1 Gy and 3.7 Gy, respectively. The EDIC values of the three SPArc plans all decreased from the level corresponding to a 50% incidence of grade 2 RIL with VMAT to that corresponding to grade 1 RIL (21).

The CTVcw in postoperative radiotherapy for left-sided breast cancer following modified radical mastectomy is characterized by the following features: 1. the target volume is superficial and adjacent to the skin; 2. the chest wall is irradiated with tangential fields; 3. the thickness of residual tissue in post-radical mastectomy patients is reduced; 4. left-sided breast cancer is located closer to the heart. These factors render it impossible to ensure adequate superficial coverage of the target volume while simultaneously protecting the OARs posterior to the target, including the heart, lungs, and spinal cord, in the absence of bolus or RS application. In our preliminary experiments, a preliminary validation was performed on SPArc plans without bolus or RS. Due to the failure to sufficiently reduce the beam energy, the range of the minimum beam energy exceeded the target depth, making it unfeasible to achieve the prescribed target coverage while ensuring OARs protection and robustness evaluation. Therefore, this plan configuration was not included in the scope of the present study.

In photon radiotherapy, deep inspiration breath-hold (DIBH) has been widely reported to significantly reduce heart and lung dose for left-sided breast cancer compared with free-breathing VMAT (23, 24). Although DIBH-VMAT provides clear dosimetric benefits, the patients in this study received VMAT under free-breathing conditions, as this remains the standard clinical practice at our institution. Multiple studies have demonstrated that, for breast cancer radiotherapy, proton therapy significantly improves target homogeneity and cardiopulmonary sparing compared with photon therapy using DIBH (25–27). While some patients receiving proton therapy may derive additional dosimetric benefits from DIBH, current evidence indicates that these benefits are generally limited and not applicable to all patients (28, 29). Therefore, DIBH should be considered an optional rather than a mandatory strategy in proton therapy. Given that the primary aim of this study was to evaluate and compare the dosimetric impact of bolus and RS in SPArc, DIBH was not included in the present analysis.

In this study, the bolus was modeled under the ideal assumption of perfect contact with the skin for dosimetric evaluation. However, in practical clinical settings, bolus application presents several limitations that may lead to discrepancies between the planned and delivered dose distributions. First, the bolus needs to be reused for 25 fractions, which may pose potential hygiene concerns. Moreover, reduced adhesiveness in subsequent fractions may lead to poor contact between the bolus and the skin, resulting in air gaps between them and thus causing a certain deviation between the actual delivered dose and the planned dose. Second, the coverage area of the bolus may be inconsistent with the preset range in the TPS, especially at the edge regions, which will induce a considerable dose discrepancy between the actual dose at the edges and the planned dose. Third, for hygiene reasons, bolus cannot be reused across different patients, which to a certain extent increases the treatment costs for patients.

The application of RS in proton therapy is expected to address these limitations. Mounted on the treatment head, RS can be reused for an extended period. After importing its model parameters into the TPS, the dose distributions of the target volume and OARs can be accurately calculated, eliminating discrepancies between the actual coverage range and the preset range in the TPS. Furthermore, compared with bolus application, RS can yield lower NTCP values for the heart and lungs, while also reducing patients’ economic burden to some extent and decreasing the workload of radiation therapists.

However, the disadvantage of the RS is that it causes an increase in spot size, which in turn leads to a reduction in dose conformity. The angular distribution of protons on the downstream surface of the RS varies with distance, resulting in geometric broadening of the beam cross-section at the patient’s body surface. Therefore, to achieve the minimum spot size, the air gap between the RS and the patient’s body surface should be minimized (15). This, however, introduces a potential risk of gantry-patient collision. Consequently, these drawbacks become more pronounced when treating tumors with complex surrounding anatomical structures, such as bilateral head and neck tumors or skull base tumors (30). For breast cancer, however, our statistics on the minimum air gap indicated that the use of RS does not significantly reduce the air gap between the treatment head and the patient. Moreover, the increased spot size induced by RS does not exert a significant impact on the target volume dose distribution; on the contrary, RS provides better protective effects for some OARs. This may be attributed to the excellent modulation capability of the SPArc technology itself on proton pencil beams, as well as the anatomical characteristics of the breast cancer target volume. Therefore, the use of a 4-cm-thick RS is completely acceptable. If further optimization of RS materials and thickness can be performed, a balance can be struck between the air gap and spot size, allowing the selection of a smaller spot size while avoiding the potential risk of gantry-patient collision.

Over the past decade, the application of proton therapy has been increasingly widespread, with continuous advancements in associated technologies. As the SPArc algorithm undergoes further optimization, the robustness of its treatment delivery will be enhanced (31). The findings of our study provide critical evidence for the clinical application of SPArc in breast cancer radiotherapy: in the absence of RS or bolus, SPArc fails to achieve a favorable balance among target volume coverage, OARs sparing, and treatment plan robustness for chest wall target volumes adjacent to the skin in the context of postoperative radiotherapy following modified radical mastectomy for breast cancer. It is therefore necessary to use bolus to compensate for superficial dose deficits or RS to modulate the spread-out Bragg peak distribution and beam distal edge position, thereby improving the target volume dose distribution. No significant advantages were observed when the two modalities were used in combination. From a clinical application perspective, the use of an RS may be preferentially considered for patients undergoing SPArc-based proton therapy after modified radical mastectomy for breast cancer. Compared with bolus, RS provides comparable or slightly improved target coverage and dose conformity, while reducing radiation exposure to the heart and lungs, thereby further lowering the NTCP of pericardial effusion and radiation pneumonitis. In addition, RS mitigates air gap issues caused by suboptimal skin contact associated with bolus application and reduces limitations related to hygiene management and inter-patient reuse. In clinical practice, these advantages translate into improved treatment quality and consistency, reduced burden on the radiotherapy workforce, and enhanced cost-effectiveness, thereby contributing to greater efficiency and sustainability of cancer care delivery.

However, this study has several limitations. First, all dosimetric analyses were based on a fixed proton RBE of 1.1, and further comprehensive evaluations incorporating biological effects—such as variations in the relative biological effectiveness at the distal edge of the proton beam and linear energy transfer (LET) optimization—are required (32). Second, this study did not systematically investigate the impacts of bolus thickness and material, as well as RS thickness and material, on dose distributions, which need to be further considered in subsequent experiments. Finally, the small sample size of cases in this study may compromise statistical power, necessitating validation with a larger sample cohort.

## 5 Conclusion

This study confirms that, in postoperative radiotherapy for left-sided breast cancer following modified radical mastectomy, the SPArc technique can significantly reduce the irradiation dose, NTCP, and EDIC delivered to OARs such as the heart and lungs, while ensuring adequate target volume coverage. All SPArc plans exhibited satisfactory robustness under range and setup uncertainties. Among the SPArc-based regimens, the standalone application of the range shifter (SPArcR) yields the most pronounced protective effects on the heart, maximally reducing the risk of pericardial effusion. Furthermore, it circumvents the limiting factors associated with the clinical use of bolus, including setup variability and hygiene concerns. In summary, SPArcR achieves an optimal balance among plan robustness, target volume coverage, OARs sparing, and clinical practicality. This study provides evidence for the selection of individualized proton therapy regimens for breast cancer; however, its clinical translation still requires validation with larger sample sizes and long-term follow-up data.

## Data Availability

The datasets generated and analyzed during this study are not publicly available due to privacy restrictions, but are available from the corresponding author upon reasonable request.

## Glossary

SPArc: spot-scanning proton arc
RS: range shifter
NTCP: normal tissue complication probability
EDIC: effective dose to immune cells
SPArcB: SPArc with bolus without RS
SPArcR: SPArc without bolus with RS
SPArcRB: SPArc with bolus with RS
WHO: World Health Organisation
IMPT: intensity-modulated proton therapy
IMRT: intensity-modulated radiotherapy
VMAT: volumetric modulated arc therapy
OARs: organs at risk
CTV: clinical target volume
CTVsc: supraclavicular lymph node clinical target volume
CTVcw: chest wall clinical target volume
PTV: planning target volume
LAD: left anterior descending artery
RBE: relative biological effectiveness
TPS: treatment planning systems
DVH: Dose–Volume Histogram
CI: conformity index
HI: homogeneity index
TD₅₀: tolerance dose for 50% complication
EUD: equivalent uniform dose
MLD: mean lung dose
MHD: mean heart dose
ITDV: integral total dose volume
LET: linear energy transfer
DIBH: deep inspiration breath-hold
